# Constraint-Guided Behavior Transformer for Centralized Coordination of Connected and Automated Vehicles at Intersections

**DOI:** 10.3390/s24165187

**Published:** 2024-08-11

**Authors:** Rui Zhao, Yuze Fan, Yun Li, Kui Wang, Fei Gao, Zhenhai Gao

**Affiliations:** 1 College of Automotive Engineering, Jilin University, Changchun 130025, China; rzhao@jlu.edu.cn (R.Z.); fanyz23@mails.jlu.edu.cn (Y.F.); gaozh@jlu.edu.cn (Z.G.); 2 Graduate School of Information and Science Technology, The University of Tokyo, Tokyo 113-8654, Japan; li-yun@g.ecc.u-tokyo.ac.jp; 3 School of Mechanical Engineering, Beijing Institute of Technology, Beijing 100081, China; 3120230321@bit.edu.cn; 4 National Key Laboratory of Automotive Chassis Integration and Bionics, Jilin University, Changchun 130025, China

**Keywords:** reinforcement learning, connected and automated vehicles, behavior transformer, constraint-guided, autonomous intersection management

## Abstract

The centralized coordination of Connected and Automated Vehicles (CAVs) at unsignalized intersections aims to enhance traffic efficiency, driving safety, and passenger comfort. Autonomous Intersection Management (AIM) systems introduce a novel approach for centralized coordination. However, existing rule-based and optimization methods often face the challenges of poor generalization and low computational efficiency when dealing with complex traffic environments and highly dynamic traffic conditions. Additionally, current Reinforcement Learning (RL)-based methods encounter difficulties around policy inference and safety. To address these issues, this study proposes Constraint-Guided Behavior Transformer for Safe Reinforcement Learning (CoBT-SRL), which uses transformers as the policy network to achieve efficient decision-making for vehicle driving behaviors. This method leverages the ability of transformers to capture long-range dependencies and improve data sample efficiency by using historical states, actions, and reward and cost returns to predict future actions. Furthermore, to enhance policy exploration performance, a sequence-level entropy regularizer is introduced to encourage policy exploration while ensuring the safety of policy updates. Simulation results indicate that CoBT-SRL exhibits stable training progress and converges effectively. CoBT-SRL outperforms other RL methods and vehicle intersection coordination schemes (VICS) based on optimal control in terms of traffic efficiency, driving safety, and passenger comfort.

## 1. Introduction

Intersections are frequent sites of accidents in urban traffic and major bottlenecks for congestion. Traditional traffic management methods often fail to effectively address these issues, especially during peak hours and in complex traffic environments. With the rapid development of artificial intelligence and wireless communication technologies, Vehicle-to-Infrastructure (V2I) technology is considered the most promising solution to these problems [1,2,3]. V2I technology enables efficient communication between vehicles and road infrastructure such as traffic signals, sensors, cameras, etc., facilitating real-time information sharing and processing. Through this technology, connected and autonomous vehicles (CAVs) within intersections can communicate in real time with road infrastructure, achieving centralized control and coordinated management of vehicles. This not only allows for collision-free driving among vehicles but also optimizes traffic flow, reduces congestion, and improves the overall efficiency of the traffic system.

Against this backdrop, Autonomous Intersection Management (AIM) systems have emerged. AIM leverages V2I technology, utilizing intelligent algorithms and real-time data processing to accurately manage and schedule traffic at intersections [4]. Through AIM, autonomous vehicles can make dynamic adjustments based on real-time traffic conditions, avoid potential collision risks, and optimize travel paths. This not only enhances traffic safety but also significantly improves traffic fluidity and efficiency, helping to resolve urban traffic bottlenecks. The current AIM coordinated control methods are mainly divided into three categories: rule-based methods, optimization-based methods, and Reinforcement Learning (RL)-based methods.

Rule-based methods constrain and schedule vehicle movements by setting traffic rules and priorities. Typical rule-based methods include First-Come First-Served (FCFS) [5], Fast First-Served (FFS) [4,6], and Longest Queue First (LQP) [7]. FCFS is a straightforward method that releases vehicles based on their arrival order; FFS, on the other hand, takes vehicle speed into consideration, prioritizing faster vehicles to improve overall traffic efficiency. The LQP method prioritizes lanes with longer queues in order to reduce long waiting times. Although these rule-based methods are simple to operate and easy to implement, they have significant limitations. When faced with complex and highly dynamic traffic conditions, these methods often fall short. For instance, FCFS can lead to long delays for certain directions under asymmetric traffic flow, failing to effectively alleviate congestion. Additionally, these methods have limited capacity to handle special situations, making it difficult to adapt to emergencies and anomalies. Moreover, in scenarios with significantly unbalanced traffic flows in different directions, some vehicles may remain stationary for extended periods due to priority constraints, thereby reducing traffic efficiency.

Optimization-based methods improve coordination by using mathematical models and optimization algorithms to allocate specific time slots or plan effective paths, thereby optimizing traffic flow and reducing intersection wait times. Bichiou et al. [8] described vehicle movement through nonlinear equations. They used a scheduler to optimize vehicle trajectories and assign time slots to each vehicle, achieving coordinated control of the intersection. Lu et al. [9] employed a Mixed Integer Programming (MIP) model to generate the fastest discrete-time trajectory for each vehicle approaching the intersection, ensuring collision-free passage in the shortest time. Kamal et al. [10] and Katrinok et al. [11] utilized Model Predictive Control (MPC) methods to optimize vehicle speed and acceleration, avoiding collisions and improving traffic flow. However, despite their ability to significantly enhance traffic flow and reduce wait times at intersections, these optimization methods have high computational complexity, making real-time control challenging in practical applications. During peak traffic periods or at complex intersections, computational demands can increase sharply, causing optimization algorithms to struggle with rapid real-time responses, which limits their practical application and efficiency.

RL-based methods achieve autonomous decision-making and coordinated control by enabling vehicles to interact with the traffic environment. These methods define state and action spaces and design reward functions to guide vehicles towards efficient and collision-free intersection passage. Abdulhai et al. [12] and Zhou et al. [13] used *Q*-learning and Deep Deterministic Policy Gradient (DDPG), respectively, to enhance traffic signal control, demonstrating the significant potential of RL in signalized intersections. Ye et al. [14] employed hierarchical action spaces and introduced a fairness index for vehicle movement as a metric, using a Deep *Q* Network (DQN) to achieve fair and efficient traffic signal control. Guan et al. [15] adopted the Model Accelerated Proximal Policy Optimization (MA-PPO) method, incorporating prior models and constraint expansion to improve sample efficiency and accelerate learning. This approach showed superior performance in centralized coordination at unsignalized intersections. Al-Sharman et al. [16] used the Soft Actor-Critic (SAC) method in the motion planning layer to train vehicles for safe yet non-conservative action at intersections, while the TD3 algorithm was employed to optimize cooperative trajectory planning at unsignalized intersections, enhancing traffic throughput while ensuring driving safety [17]. However, current RL-based AIM methods face challenges in model reasoning capabilities and robust safety assurance, making it difficult to maintain traffic efficiency and safety in highly complex and stochastic intersections.

To address the challenge of insufficient model reasoning capabilities in RL, transformers have been introduced due to their outstanding performance, generalization ability, and scalability [18]. Transformer models are based on attention mechanisms. They possess strong sequence modeling capabilities and are able to capture long-distance dependencies, making them suitable for handling complex temporal data. The introduction of transformers has provided new insights for applying RL in complex environments. Zambaldi et al. [19] integrated transformers into RL using multi-head dot-product attention to achieve relational reasoning in structured observations, significantly enhancing the application of attention mechanisms in complex RL environments. However, the unique demands of RL training, including alternating data collection and policy optimization, present challenges for transformer applications, including instability due to changing data distributions and sensitivity to design choices. Offline RL has emerged as a transformative approach to address these challenges, solving RL problems using large-scale experience datasets and redefining transformer applications as sequence modeling problems. This method allows transformers to leverage their sequence modeling capabilities without the need to discount future rewards. Noteworthy advances include the Decision Transformer (DT) by Chen et al. [20], which predicts actions based on past states, actions, and rewards, and the Trajectory Transformer (TT) by Janner et al. [21], which uses beam search to predict the optimal action based on past states, actions, and rewards. However, TT models perform poorly on tasks requiring high real-time responsiveness due to the time-intensive nature of beam search. Further innovations, such as the Generalized Decision Transformer (GDT) by Furuta et al. [22] and the *Q*-learning Decision Transformer (QDT) by Yamagata et al. [23], have introduced methods such as hindsight information matching and dynamic programming, enhancing the practical utility of transformers in RL. Nonetheless, despite these advances offline RL may be constrained by the quality of the training dataset, potentially leading to suboptimal policy. To address this issue, Zheng et al. [24] proposed the Online Decision Transformer (ODT), combining offline RL with online fine-tuning. However, ODT relies on supervised learning to minimize the difference between actions collected online and expert data, potentially limiting the model’s ability to explore high-reward actions and achieve robust policy updates. Additionally, learning safe policy from experience datasets presents a significant challenge when applying transformers to offline RL. To address these safety concerns, Liu et al. [25] adopted a Multi-Objective Optimization (MOO) approach in offline RL to maximize rewards while ensuring safety constraints. Zhang et al. [26] proposed SaFormer, which restricts the action space with cost-related labels and enforces safety through a posterior validation step.

Ensuring safety in the application of RL to AIM is a crucial concern, and Safe RL has emerged as a solution to this challenge. Constrained Markov Decision Processes (CMDP) are employed to address Safe RL issues by introducing safety constraints. These constraints ensure that the model not only maximizes rewards but also adheres to optimal policies within the defined safety limits. Various model-based and model-free methods have been developed to tackle CMDP problems, including primal–dual methods, trust region methods with safety constraints, Lyapunov-based methods, and Gaussian process optimization [27,28,29,30,31,32,33,34,35]. Additionally, algorithms such as PID-based Lagrangian methods and “SauteRL” have been designed to enhance safety during the learning process by using state augmentation and penalty adjustments. Other approaches, such as “SimmerRL”, dynamically adjust safety thresholds to improve policy robustness and safety. Model-based Safe RL methods leverage environmental models to predict future states and rewards, thereby improving learning efficiency and safety [36,37,38,39]. However, constructing accurate models in complex large-scale environments such as intelligent traffic systems remains challenging, limiting the applicability of model-based Safe RL methods in AIM systems. While model-free methods do not require environmental models and directly learn policies through interaction with the environment, existing RL methods often struggle to solve autonomous intersection management problems due to insufficient inference capabilities, making it difficult to cope with complex and highly stochastic traffic environments.

To address the challenges of insufficient model reasoning capabilities and difficulty in ensuring robust safety in RL, in this research we propose the Constraint-Guided Behavior Transformer Safe Reinforcement Learning (CoBT-SRL) method. This Safe RL approach first involves collecting a large amount of historical trajectory data using expert policy. Specifically, these trajectory data include information such as states, actions, rewards, and costs. These data undergo rigorous collection and filtering processes to ensure their quality and reliability. In the AIM dataset, each data point includes a past state and action along with the associated future Reward to Go (RTG) and Cost to Go (CTG). The RTG represents the total reward obtainable from the current state over a future period, while the CTG represents the potential total cost over the same period. Next, an autoregressive modeling approach is used, taking these historical data as input to predict future actions. In this process, a transformer model is introduced to leverage its powerful capabilities in sequence modeling. The transformer model excels at capturing long-range dependencies and effectively handling and modeling complex temporal data. This approach enhances data efficiency while ensuring robust safety, thereby improving generalization to highly complex and stochastic traffic environments.

The main contributions of this study are as follows:

(1) This study proposes CoBT-SRL, utilizing a transformer as a policy to achieve sequence decision-making for vehicle driving behavior. This method leverages historical states, actions, RTG, and CTG to predict future actions, taking advantage of the transformer’s ability to capture long-range dependencies and strong reasoning capabilities while improving data sample utilization efficiency and safety.
(2) This study models the AIM system as a continuous task rather than a discrete one. By maintaining and dynamically updating the real-time status information of CAVs, the system can better simulate and respond to dynamic conditions in real-world environments. This continuous task modeling approach not only enhances the realism of simulations but also significantly improves the generalization ability of RL-based AIM policy under various traffic densities and scenarios at unsignalized intersections.
(3) The effectiveness of the model was validated through extensive simulation experiments. The results indicate that the CoBT-SRL training process is stable and converges well. During testing, CoBT-SRL was compared with advanced RL methods such as PPO and TD3 as well as the optimal control-based Vehicle Intersection Coordination Scheme (VICS). CoBT-SRL achieved superior results in terms of traffic efficiency, driving safety, and passenger comfort.

The rest of this paper is structured as follows: Section 2 defines the problem and describes the framework of this study; Section 3 introduces the modeling of the AIM problem within the CMDP framework, defining the state space and action space and then designing the reward and cost functions; Section 4 discusses the methodology for reinforcement learning; Section 5 details the experimental setup and analysis of the results; finally, Section 6 summarizes the work and looks forward to future research directions.

## 2. Problem Definition and Method Framework

### 2.1. Problem Definition

The intersection scenario studied in this paper involves a typical unsignalized four-way intersection with a multi-lane road layout. Each inbound lane is designed to allow vehicles to go straight, turn left, or turn right as needed. As vehicles gradually approach the intersection from a distance, they move from an uncontrolled area into a controlled area. Within this controlled area, the AIM system monitors and controls all approaching vehicles in real time. Advanced sensor and communication technologies continuously obtain critical data for each vehicle, including real-time position, speed, and driving direction. Based on these data, the system dynamically adjusts the driving routes and speeds of the vehicles to ensure safe and efficient passage through the intersection. Utilizing complex algorithms and decision models, the system optimizes the sequence and paths of vehicles in real time to minimize traffic conflicts and congestion to the greatest extent possible.

Each vehicle *i* periodically sends its dynamic driving data $s_{i}=\left[x_{i},y_{i},v_{i},\beta_{i},\zeta_{i}\right]$ to the AIM system via V2I wireless communication. In these data, $x_{i}$ and $y_{i}$ represent the real-time positional coordinates of vehicle *i*, providing precise spatial location information; $v_{i}$ represents the current driving speed of the vehicle, reflecting its motion state; $\beta_{i}$ indicates the intended driving direction at the intersection (left turn, straight, or right turn); and $\zeta_{i}$ is the positional encoding information of the vehicle on the road, which is used for localization and trajectory planning.

The AIM system uses these data to control the driving behavior of each vehicle in real time through an RL algorithm. Based on the traffic conditions and the state of each vehicle, the system dynamically adjusts the target speed $v_{i}^{goal}$ and target steering angle $\varphi_{i}^{goal}$ to ensure efficient and orderly traffic flow at the intersection. This approach enhances overall traffic efficiency and improves passenger comfort while ensuring driving safety. At the vehicle motion control level, a PID controller is employed to achieve precise longitudinal and lateral control. The PID controller calculates the required throttle, braking force, and steering wheel angle based on the target speed and steering angle. By adjusting these parameters, the vehicle can smoothly and safely accelerate, decelerate, and turn, thereby achieving precise motion control and ensuring that the vehicle follows the predetermined trajectory.

The problem is defined as follows: at each time step, given the static road information of the intersection (i.e., road topology and geometry) and the dynamic state information of all vehicles. including position, speed, driving intention, and positional encoding information, the CoBT-SRL-based AIM system determines the desired speed and steering angle of vehicles within the control area in real time. This coordination aims to achieve collision-free and efficient traffic flow for all vehicles at the intersection.

### 2.2. Method Framework

**Figure 1:**
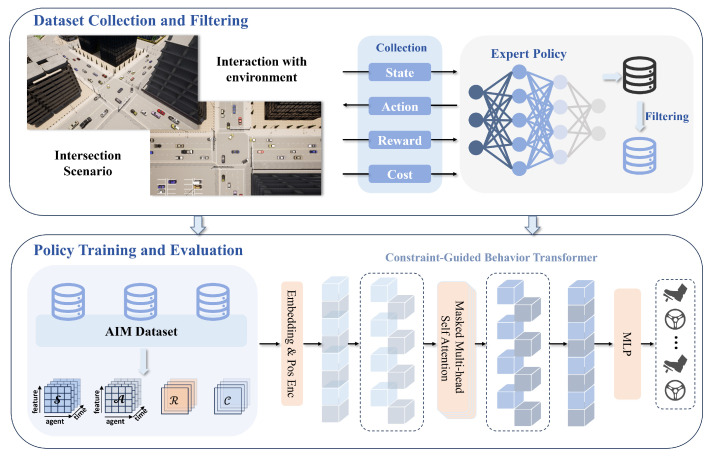
The proposed CoBT-SRL framework is primarily divided into two components: (1) Data Collection and Filtering, and (2) Policy Training and Evaluation. Historical trajectory information is collected by interacting with the environment using expert policy, and the collected data are filtered. Then, a transformer model is used as the policy for training and evaluation.

The CoBT-SRL framework proposed in this study is divided into two main components, as shown in Figure 1: (1) Dataset Collection and Filtering, and (2) Policy Training and Evaluation. The CoBT-SRL method aims to address challenges related to low data efficiency, insufficient model reasoning capabilities, and difficulties in ensuring driving safety.

In the dataset collection and filtering phase, historical trajectory information is obtained through interactions between the existing policy and the online environment, forming an expert dataset. This dataset includes past global states, joint actions, reward and cost returns, and other relevant information. The collected data are then filtered to further enhance the quality of the dataset. The filtered expert dataset is used for subsequent policy training and evaluation.

During the policy training and evaluation phase, a transformer model is employed as the policy network to predict future actions based on historical states, actions, RTG, and CTG. The transformer’s ability to capture long-range dependencies improves data sample utilization efficiency and provides robust safety. Through policy training and evaluation, a more robust policy can be obtained for the AIM system that enhances traffic efficiency, driving safety, and passenger comfort.

## 3. Safe Reinforcement Learning for Autonomous Intersection Management

### 3.1. Safe RL Formulation

To address the challenges of Safe RL, the traditional Markov Decision Process (MDP) framework is extended by introducing additional safety constraints, resulting in a CMDP model. This CMDP framework explicitly considers safety factors during the decision-making process, providing a foundation for solving the complexity of Safe RL problems. The CMDP model is defined as a tuple $\left\{S,A,P,r,c,d,\rho^{0},\gamma \right\}$, where S→R represents the state space, encompassing any state the agent acquires from the environment; A→R represents the action space, encompassing all possible actions the agent can perform; and P:S×A×S→[0,1] represents the transition probability distribution, describing the probability of transitioning from state $s_{t}\in S$ to the next state $s_{t+1}\in S$ after executing action $a_{t}\in A$ at time step *t*. The reward function r:S×A×S→R represents the immediate reward obtained after transitioning from state $s_{t}$ to state $s_{t+1}$ by performing action $a_{t}$. The set of cost functions $c={\left\{c_{i}\right\}}_{i=1}^{N_{c}}$ defines specific environmental safety risk constraints, with each cost function $c_{i}:S\times A\times S\to R$ mapping the transition tuple to a safety cost. Here, $N_{c}$ represents the number of different safety constraints considered, with $N_{c}$ distinct cost functions $c_{i}$ in the set c. The threshold $d={\left\{d_{i}\right\}}_{i=1}^{N_{c}}$ associated with each cost function represents the maximum allowable cost to ensure safety, while $\rho^{0}$ represents the initial state distribution describing the probability distribution of states at the start of the process and γ∈[0,1) is the discount factor indicating the extent to which future rewards and costs are considered during decision-making.

According to the CMDP model, the agent interacts with the environment in discrete time steps. At each time step *t*, the agent receives the current state $s_{t}\in S$ from the environment and executes an action $a_{t}∼\pi \left(\cdot |s_{t}\right)$ based on the policy π. After performing action $a_{t}$, the agent receives a reward $r=r(s_{t},a_{t},s_{t+1})$ and a set of costs $c={\left\{c_{i}\left(s_{t},a_{t},s_{t+1}\right)\right\}}_{i=1}^{N_{c}}$. The environment then transitions to a new state $s_{t+1}∼P\left(\cdot |s_{t},a_{t}\right)$.

The interactions between all agents and the environment continuously generate trajectories, forming a complete trajectory $\tau ={\left\{s_{t},a_{t},r_{t},c_{t}\right\}}_{1}^{N_{t}}$, where $N_{t}$ represents the number of time steps in an episode. In each trajectory, the RTG and CTG are defined as

(1) $$R_{T_{current}}=\sum_{t=T_{current}}^{N_{t}}r_{t},\;C_{T_{current}}=\sum_{t=T_{current}}^{N_{t}}c_{t},$$

where $T_{current}$ represents the current time step. These metrics indicate the expected return and cost from the current time *t* to the end of the trajectory. They provide crucial insights for the CMDP model and help the agent to evaluate future rewards and costs to optimize actions under specified constraints, thereby maximizing rewards while adhering to cost constraints.

### 3.2. State Space and Action Space

In the Safe RL-based AIM system, designing a reasonable state space is crucial for the learning process. A well-designed global state space allows each vehicle to consider its own state and action, understand and predict the action of other vehicles, and grasp the overall dynamics of the environment. Unlike previous RL-based AIM problems that only consider fixed vehicles at intersections, this study focuses on continuous and stochastic traffic flow at intersections. In this context, randomly arriving vehicles more realistically simulate actual traffic flow, prompting the model to learn more robust, adaptable, and generalizable policy, thereby enhancing its ability to handle highly complex and stochastic real-world scenarios. Additionally, designing a reasonable observation space in a dynamic environment is essential for the model to effectively understand the random variations in traffic scenarios and make more flexible and efficient decisions.

To effectively address the complex and dynamically changing intersection scenarios, this study designs an efficient global state space that includes temporal information. These states are observed at different time points and influence vehicle decisions, thereby better reflecting the historical trajectories of vehicle states and their interactions with the environment. Moreover, the global state space at each moment is defined as the ordered concatenation of all vehicle states in order to promote the coordination efficiency of the AIM system. Based on this design, for a vehicle *i* at the current specific time step $T_{current}$, the input state can be represented as ${\left\{S_{t}^{i}\right\}}_{t=T_{current}-K}^{t=T_{current}}$, where *K* represents the context length. Each vehicle’s state space includes its current speed information $v_{t}^{i}$, distance to the target point $d_{t}^{i}$, steering intention $\beta_{t}^{i}$, and lane position information $\zeta_{t}^{i}$:

(2) $$S_{t}^{i}=\left\{v_{t}^{i},d_{t}^{i},\beta_{t}^{i},\zeta_{t}^{i}|i\in N_{a},t\in N_{t}\right\}$$

where $N_{a}$ represents the total number of vehicles considered in the scenario and the speed information $v_{t}^{i}$ represents the real-time speed of vehicle *i* at time step *t*. The AIM system determines the vehicle’s target position at the intersection exit based on the current vehicle’s steering intention $\beta_{t}^{i}$ and road encoding $\zeta_{t}^{i}$. Subsequently, the target distance $d_{t}^{i}$ between the vehicle and the target position can be calculated based on the specific trajectory. Notably, in order to enhance the efficiency of neural network learning and strengthen the representation of state space features, the steering intention and road encoding information of the vehicle are represented using one-hot encoding.

By designing a global state space that considers the temporal and dynamic characteristics of real intersection scenarios, the complex state space fully leverages the transformers mechanism to capture global attention as policy. This maps the global state to efficient and reasonable joint actions. The action output is defined as the action set for each vehicle, represented as ${\left\{A_{t}^{i}\right\}}_{t=T_{current}}^{t=T_{current}+K}$. Similarly, future actions over multiple time steps are used as outputs to help the model with long-term planning and adjustment. Specifically, for each vehicle, the speed *v* and steering angle φ are used as action outputs to account for lateral and longitudinal motion control, represented as

(3) $$A_{t}^{i,goal}=\left\{v_{t}^{i,goal},\varphi_{t}^{i,goal}|i\in N_{a},t\in N_{t}\right\}.$$

Furthermore, the motion control layer of each vehicle utilizes a PID controller to compute the required throttle, brake force, and steering wheel for the vehicle’s motion, enabling longitudinal and lateral control. Specifically, the longitudinal and lateral control errors $e_{t}^{i,lon}$ and $e_{t}^{i,lat}$ are calculated as follows:

(4) $$e_{t}^{i,lon}=v_{t}^{i,goal}-v_{t}^{i}$$

(5) $$e_{t}^{i,lat}=\arccos\left(\frac{{\vec{\omega }}_{t}^{i}\cdot {\vec{v}}_{t}^{i}}{∥{\vec{\omega }}_{t}^{i}∥∥{\vec{v}}_{t}^{i}∥}\right)$$

where $v_{t}^{i,goal}$ and $v_{t}^{i}$ represent the desired velocity and current velocity of vehicle *i*, respectively, ${\vec{v}}_{t}^{i}$ represents the velocity vector of vehicle *i*, and ${\vec{\omega }}_{t}^{i}$ represents the vector from the current position of vehicle *i* to its target position. Then, the longitudinal control input $u_{t}^{i,lon}$ and lateral control input $u_{t}^{i,lat}$ are computed separately using PID controllers:

(6) $$u_{t}^{i,lon}=k_{p}^{lon}\cdot e_{t}^{i,lon}+k_{i}^{lon}\cdot \int_{0}^{t}e_{u}^{i,lon}\;du+k_{d}^{lon}\cdot \frac{d}{dt}e_{t}^{i,lon}$$

(7) $$u_{t}^{i,lat}=k_{p}^{lat}\cdot e_{t}^{i,lat}+k_{i}^{lat}\cdot \int_{0}^{t}e_{u}^{i,lat}\;du+k_{d}^{lat}\cdot \frac{d}{dt}e_{t}^{i,lat}$$

where $k_{p}^{lon}$,$k_{i}^{lon}$,$k_{d}^{lon}$ and $k_{p}^{lat}$,$k_{i}^{lat}$,$k_{d}^{lat}$ represent the coefficients of the proportional, integral, and derivative terms in the longitudinal and lateral control, respectively.

### 3.3. Reward and Cost Functions

In RL, reward functions and cost functions are crucial for guiding the agent to learn effective policy. For traditional tasks, maximizing rewards typically directs the agent to learn optimal policy, while penalizing undesirable actions provides negative feedback. However, in autonomous driving this approach may lead to vehicles simply avoiding any actions that might incur penalties, resulting in overly conservative driving behavior and reduced efficiency of the learned policy. By introducing cost functions for multi-objective optimization, vehicles can find an appropriate balance between efficiency, comfort, and adherence to safety constraints, rather than merely avoiding actions that might incur penalties. By combining reward functions and cost functions, they jointly guide the learning behavior of the policy, ensuring that vehicles in the AIM system can drive efficiently, safely, and comfortably.

For traditional AIM systems with single scenarios and simple tasks, using a specific reward structure can accelerate the learning efficiency of the policy. However, this approach may also lead to overfitting and difficulties in adapting to new environments. In contrast, for complex intersection scenarios with randomly arriving vehicles, a comprehensive and careful consideration of reward design is necessary to enhance the generalization ability and robustness of the policy. Specifically, the designed reward function $R_{CoBT-SRL}$ comprehensively considers traffic efficiency, safety, and comfort, combining both sparse and dense rewards, and is divided into three main components, represented as

(8) $$R_{CoBT-SRL}=R_{efficiency}+R_{comfort}+R_{safety}.$$

The efficiency term $R_{efficiency}$ measures vehicle passage metrics within a fixed timestep. It considers two aspects, traffic throughput and average speed, represented as shown below.

(9) $$R_{{efficiency}_{1}}=\left\{\begin{array}{cc} \delta_{1} & \mathrm{if}\;\mathrm{successfully}\;\mathrm{passed}\\ 0 & \mathrm{otherwise} \end{array}\right.$$

(10) $$R_{{efficiency}_{2}}=\omega_{1}\cdot {\bar{v}}_{t}$$

The parameter $\delta_{1}$ is a fixed reward for each vehicle that successfully passes through the traffic system, representing the system’s goal of maintaining high throughput. The weight coefficient $\omega_{1}$ determines the importance of average speed in the reward function, allowing the system to balance throughput and speed depending on the traffic management objectives. The average speed of all vehicles at time *t*, denoted as ${\bar{v}}_{t}$, is calculated as ${\bar{v}}_{t}=\frac{1}{N_{a}}\sum_{i=1}^{N_{a}}v_{t}^{i}$, where $N_{a}$ is the number of vehicles and $v_{t}^{i}$ is the individual speed of vehicle *i*. Compared to the total sum of speeds, the average speed better reflects real-time traffic flow, preventing scenarios in which the high speed of a few vehicles skews the perception of traffic conditions and promoting a smooth and efficient overall flow of traffic.

Subsequently, the comfort term $R_{comfort}$ is designed by considering the smoothness of vehicle movement, considering acceleration and jerk. represented as follows:

(11) $$R_{comfort}=\omega_{2}\cdot a_{t}^{i}+\omega_{3}\cdot j_{t}^{i}$$

where $\omega_{2}$ and $\omega_{3}$ are weight coefficients that reflect the relative importance of acceleration and jerk in the comfort evaluation. These parameters can be adjusted to prioritize different aspects of comfort, such as minimizing acceleration to avoid passenger discomfort or reducing jerk to ensure smoother transitions. Here, $a_{t}^{i}$ and $j_{t}^{i}$ represent the acceleration and jerk (rate of change of acceleration) of vehicle *i* at time *t*, respectively. Acceleration is calculated as $a_{t}^{i}=\frac{v_{t}^{i}-v_{t-1}^{i}}{\Delta t}$, while jerk is calculated as $j_{t}^{i}=\frac{a_{t}^{i}-a_{t-1}^{i}}{\Delta t}$. Smooth traffic flow typically requires vehicles to maintain relatively constant speeds while avoiding frequent acceleration or deceleration, thereby ensuring overall traffic smoothness and passenger comfort. Finally, considering the importance of safety, the negative value of the cost function is included as a penalty term in the reward function, specifically as

(12) $$R_{safety}=-C_{CoBT-SRL}.$$

In order to effectively ensure the safety of the AIM system, the cost function primarily considers whether the Distance to Collision (DTC) between vehicles is maintained stably, whether the Time to Collision (TTC) between vehicles at risk of collision satisfies constraints, and the cost that is incurred in the event of a collision. This can be represented as

(13) $$C_{CoBT-SRL}=C_{dtc}+C_{ttc}+C_{collision},$$

where $C_{dtc}$ is used to measure the risk between two vehicles when the relative distance $dtc^{i,i^{\prime }}$ is less than a certain threshold. The cost function is increased to quantify the risk at that moment, which can be represented as shown below.

(14) $$C_{\mathrm{dtc}}=\left\{\begin{array}{cc} \delta_{2} & \mathrm{if}\;dtc^{i,i^{\prime }}<DTC^{\mathrm{threshold}}\\ 0 & \mathrm{otherwise} \end{array}\right.$$

This design increases the costs as the distance between vehicles decreases, encouraging vehicles to maintain safe distances more accurately. By dynamically adjusting the distance threshold $DTC^{\mathrm{threshold}}$, the penalty intensity can be flexibly set according to different traffic scenarios and safety requirements. Similarly, for vehicles with potential conflict points, $TTC^{\mathrm{threshold}}$ represents their respective TTC thresholds. The cost function $C_{ttc}$ is designed as shown below.

(15) $$C_{\mathrm{ttc}}=\left\{\begin{array}{cc} \delta_{3} & \mathrm{if}\;ttc^{i,i^{\prime }}<TTC^{\mathrm{threshold}}\\ 0 & \mathrm{otherwise} \end{array}\right.$$

**Figure 2:**
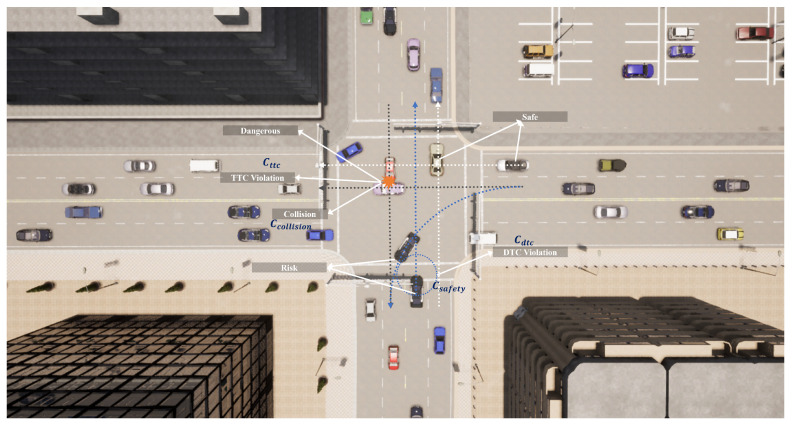
Consideration of safety cost sub-items and trajectories with varying risk levels at intersection: the white dashed lines indicate safe trajectories, the blue dashed lines potentially risky trajectories, and the black dashed lines highly dangerous trajectories.

By combining the comprehensive effects of $C_{dtc}$ and $C_{ttc}$, the AIM system achieves thorough safety risk assessment, accurately predicting collision risks and enabling the system to respond flexibly in various situations. For example, in low-speed or congested scenarios, the distance cost is emphasized, whereas under high-speed conditions the TTC penalty better reflects collision risks. Additionally, a significant penalty is imposed if a collision occurs between vehicles, helping to guide vehicles away from behaviors that might lead to collisions, as shown in Figure 2. This can be represented as shown below.

(16) $$C_{\mathrm{collision}}=\left\{\begin{array}{cc} \delta_{4} & \mathrm{if}\;\mathrm{collision}\\ 0 & \mathrm{otherwise} \end{array}\right.$$

The cost function $C_{CoBT-SRL}$ consists of three subcomponents, each associated with immediate costs represented by $\delta_{2}$, $\delta_{3}$, and $\delta_{4}$. Among these, $C_{dtc}$ and $C_{ttc}$ are seen as preventive measures aimed at mitigating behaviors that increase the risk of collisions, $C_{dtc}$ is focused on maintaining static safety boundaries, and $C_{ttc}$ carries out dynamic risk assessment to address collision risk prevention at various levels. In contrast, $C_{collision}$ directly responds to collisions that have already occurred, clearly indicating that such behaviors lead to unacceptable outcomes. The parameters $\delta_{2}$, $\delta_{3}$, and $\delta_{4}$ are chosen to appropriately weigh the importance of different safety considerations, reflecting the severity and priority of each component in the overall safety strategy. Specifically, $\delta_{2}$ might be set higher if maintaining static safety boundaries is prioritized, while $\delta_{3}$ could be more significant when dynamic risk assessments are critical; $\delta_{4}$ should typically have a large value in order to heavily penalize actual collisions, as they represent the most severe safety breach. Together, these components form a multi-level safety protection system designed to minimize collision risks in complex traffic environments by preventing and penalizing potential dangerous behaviors across different dimensions and stages, thereby enhancing the overall safety of the AIM system.

## 4. Constraint-Guided Behavior Transformer for Safe Reinforcement Learning

### 4.1. Dataset Collection and Filtering

When training a transformer model through autoregressive modeling, the quality of the dataset is crucial for the model’s performance. Collecting high-quality datasets in the real world is time-consuming and expensive, requiring significant investments in terms of time, resources, and logistics. To address this issue and maximize the utilization of trajectory samples, a dataset was constructed using historical data generated from an optimized expert policy $\pi_{expert}$ from a previous work.

Specifically, the details of dataset collection and filtering are shown in the first half of Algorithm 1. First, a sample of the initial global state $s_{1}$ is obtained in an online environment. Then, joint actions $a_{t}$ are generated and executed in the environment using the expert policy, resulting in the next state $s_{t+1}$ (lines 1–7). Meanwhile, the reward $r_{t}$ and cost $c_{t}$ for the current time step are calculated using the reward function R and cost function C. This process is repeated for the specified number of time steps $T_{l}$ to obtain a complete trajectory $\tau ={\left\{s_{t},a_{t},r_{t},c_{t}\right\}}_{t=1}^{T_{l}}$ (lines 8–11). Subsequently, a data augmentation process is performed by summing the costs in the trajectory and checking whether they are below the initially set cost constraint threshold *d* (lines 12). If the cost constraints are met, then the RTG and CTG are calculated as new labels for subsequent model input. Finally, the filtered and processed trajectory $\tau ={\left\{R_{t},C_{t},s_{t},a_{t}\right\}}_{t=1}^{T_{l}}$ is added to the dataset T. This process iterates continuously to obtain the final AIM dataset T (lines 13–17).
**Algorithm 1** CoBT-SRL for AIM**Require:** expert policy $\pi_{expert}$, trajectory samples $T_{s}$, trajectory length $T_{l}$, cost limit *d*, target entropy β, batch size *B*, learning rate $\alpha_{\theta }$, training iterations *I*, context length *K* 1:**for** each sample *s* in $T_{s}$ **do** 2:      Sample the initial global state $s_{1}$ from online env 3:      Initialize a new trajectory τ={} 4:      **for** each timestep *t* in $T_{l}$ **do** 5:            Get predicted action based on $\pi_{expert}$ 6:            $a_{t}^{i}∼\pi_{expert}\left(\cdot |s=s_{t}\right)$ 7:            Execute the joint action $a_{t}^{i}$ and get $s_{t+1}$ from online env 8:            Calculate $r_{t}$ and $c_{t}$ based on reward function and cost function 9:            $r_{t}=R\left(s_{t},a_{t},s_{t+1}\right),c_{t}=C\left(s_{t},a_{t},s_{t+1}\right)$10:            Append the new tokens to the trajectory $\tau ={\left\{s_{k},a_{k},r_{k},c_{k}\right\}}_{1}^{t-1}\cup \left\{s_{t},a_{t},r_{t},c_{t}\right\}$11:      **end for**12:      **if** $\sum_{i=1}^{T_{l}}c_{i}<d$ **then**13:            Compute RTG and CTG $R_{T_{current}}=\sum_{t=T_{current}}^{T_{l}}r_{t},\;C_{T_{current}}=\sum_{t=T_{current}}^{T_{l}}c_{t}$14:            Get the new trajectory $\tau ={\left\{R_{t},C_{t},s_{t},a_{t}\right\}}_{1}^{T_{l}}$15:      **end if**16:      Append the new trajectory τ to the AIM dataset T17:**end for**18:**for** each iteration *l* in *I* **do**19:      Sample trajectories {τ} from AIM dataset T20:      **for** each τ in {τ} **do**21:            Sample a batch of sequence of length *K*22:            $B={\left\{R_{-K,t}^{n},C_{-K,t}^{n},s_{-K,t}^{n},a_{-K,t}^{n}\right\}}_{n=1}^{B}∼\tau$23:            Predict joint action of vehicles ${\hat{a}}_{t}$24:            ${\hat{a}}_{t}∼N\left(\mu_{\theta },\Sigma_{\theta }|{\left\{R_{t},C_{t},s_{t},a_{t}\right\}}_{T_{current}-K}^{T_{current}-1}\cup \left\{R_{T_{current}},C_{T_{current}},s_{T_{current}}\right\}\right)$25:            Compute NLL loss and entropy and construct the Lagrangian function26:            L(θ,λ)=J(θ)+λ(β−H(θ))27:            Update policy parameter θ28:            $\theta \leftarrow \theta -\alpha_{\theta }\nabla_{\theta }L\left(\theta ,\lambda \right)$29:      **end for**30:**end for**

The dataset was further refined by filtering and extracting optimized data based on the cost values in the trajectories, allowing the training process to benefit from a diverse set of trajectories and promoting the development of a robust foundational policy. This approach reduces the need for expensive real-world data collection while still providing a comprehensive training dataset. Utilizing these optimized historical trajectories ensures that the autoregressive modeling process is based on realistic high-quality data, enhancing the robustness and adaptability of the policy when applied to new and unseen traffic scenarios.

### 4.2. Policy Training and Evaluation

**Figure 3:**
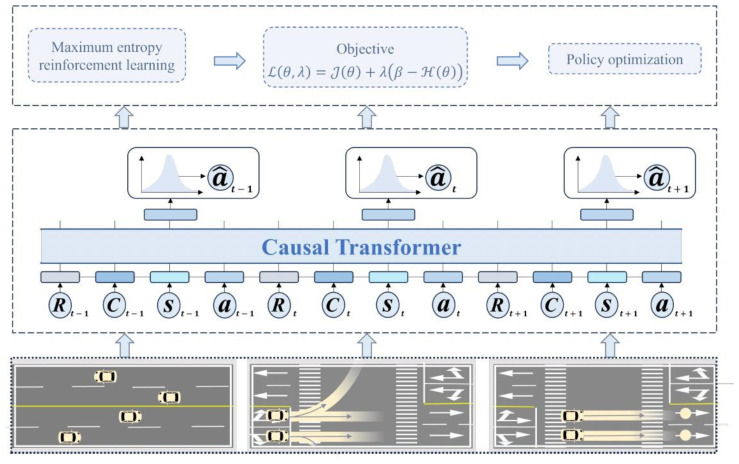
Methodology of CoBT-SRL. Using a causal transformer as the policy, historical states, actions, rewards, and cost return information obtained from the traffic environment are input for sequence modeling. This approach facilitates sequential decision-making and predicts future actions. The policy is then updated using the maximum entropy RL method, ensuring the accuracy of action outputs while enhancing the exploration capability of the policy.

The above data collection and filtering process resulted in a high-quality expert dataset T for used in RL training of a transformer-based autoregressive model. In this phase, the precollected data enable the learned policy to generalize effectively across various environments without the need for real-time interaction. This section introduces the sequence modeling for the AIM dataset, focusing on the trajectory τ in the dataset T, as shown in Figure 3. The trajectory τ is represented as shown below.

(17) $$\begin{array}{c} \tau =(R_{1},C_{1},s_{1},a_{1},R_{2},C_{2},s_{2},a_{2},\ldots ,R_{N_{t}},C_{N_{t}},s_{N_{t}},a_{N_{t}}) \end{array}$$

CoBT-SRL uses historical global states, joint actions, RTG, and CTG to predict current actions, achieving the goal of sequential decision-making. Relying solely on RTG to represent expected returns is insufficient for safety-critical tasks such as autonomous driving. CoBT-SRL aims to not only maximize cumulative returns but also to adhere to predefined cost constraints. The CTG metric is indispensable, as it represents the allowable cost from the current time step $T_{current}$ to the end of the trajectory, playing a crucial role in integrating safety constraints into action selection. Unlike traditional RL methods, CoBT-SRL constructs its model input as ${\left\{R_{t},C_{t},s_{t},a_{t}\right\}}_{T_{current}-K}^{T_{current}-1}\cup \left\{R_{T_{current}},C_{T_{current}},s_{T_{current}}\right\}$ to achieve sequential decision-making through sequence modeling.

When predicting actions, CoBT-SRL employs a stochastic policy, modeling the action distribution through a multivariate Gaussian distribution with a diagonal covariance matrix. This approach aims to enhance policy adaptability by maximizing the likelihood of actions within the dataset while introducing the flexibility to respond to different environmental conditions. The policy and its parameters are denoted by π and θ, with the mean vector represented by $\mu_{\theta }$ and the covariance matrix by $\Sigma_{\theta }$. This setup allows the stochastic policy to dynamically adjust to the decision-making environment, as detailed in Figure 3.

(18) $$\begin{array}{cc} {\hat{a}}_{t}∼N(\mu_{\theta },\Sigma_{\theta }| & {\left\{R_{t},C_{t},s_{t},a_{t}\right\}}_{T_{current}-K}^{T_{current}-1}\cup \left\{R_{T_{current}},C_{T_{current}},s_{T_{current}}\right\}) \end{array}$$

Compared to deterministic policy, stochastic policy offers significant advantages. By introducing randomness in action selection, stochastic policy facilitates the exploration of environmental performance limits rather than merely executing known optimal actions. This approach avoids local optima while enhancing exploration capabilities. Conversely, deterministic policy is prone to out-of-distribution (OOD) actions due to system bias. By varying actions in similar states, stochastic policy enables agents to develop more robust policy, thereby enhancing the generalization capability of the policy.

To quantify the consistency between the actions predicted by the stochastic policy and the actual benchmark actions, the negative log-likelihood (NLL) loss function is used.

(19) $$\begin{array}{cc} J(\theta )= & -\frac{1}{K+1}E_{T}\sum_{k=0}^{K}\left[\log\pi_{\theta }(a_{T_{current}-k}|{\left\{R_{t},C_{t},s_{t},a_{t}\right\}}_{T_{current}-K}^{T_{current}-1-k}\cup \right.\\ \; & \left.\{R_{T_{current}-k},C_{T_{current}-k},s_{T_{current}-k}\})\right] \end{array}$$

The policy update process focuses on minimizing NLL loss. However, concentrating solely on this metric may limit exploratory behavior and increase the risk of overfitting. To mitigate these issues, the Shannon entropy H(θ) of the policy $\pi_{\theta }$ is integrated into the optimization framework [40]. This approach is a hallmark of maximum entropy RL algorithms, which aim not only to minimize prediction error but also to maximize entropy. In this way, the policy is encouraged to explore a more diverse range of actions, thereby enhancing its robustness and adaptability. This dual focus on entropy maximization and loss minimization ensures a more balanced learning approach. The entropy H(θ) can be calculated as shown below.

(20) $$\begin{array}{cc} H(\theta )= & \frac{1}{K+1}E_{T}\sum_{k=0}^{K}H\left[\pi_{\theta }\left(a_{T_{current}-k}|\{R_{t},C_{t},s_{t},\right.a_{t}{\}}_{T_{current}-K}^{T_{current}-1-k}\cup \right.\\ \; & \left.\{R_{T_{current}-k},C_{T_{current}-k},s_{T_{current}-k}\}\right] \end{array}$$

The objective of the policy update is to minimize the NLL loss while ensuring that the policy’s entropy H(θ) meets or exceeds a predefined lower bound β. This approach not only improves the accuracy of the policy but also ensures an adequate level of exploration. By setting a lower entropy bound, the policy is encouraged to explore a broader range of behaviors, thereby avoiding premature convergence to suboptimal solutions. This method promotes a balanced development of exploration and exploitation, which is crucial for effective learning in complex environments. This objective can be expressed as follows:

(21) $$\begin{array}{c} \min_{\theta }J\left(\theta \right)\;s.t.\;H\left(\theta \right)\geq \beta . \end{array}$$

To tackle the objective problem characterized by the aforementioned inequality constraint described in Equation (21), the Lagrangian function for the primal problem is constructed as follows:

(22) $$\begin{array}{c} L(\theta ,\lambda )=J(\theta )+\lambda (\beta -H(\theta )) \end{array}$$

where λ∈[0,+∞) represents the Lagrange multiplier (i.e., the dual variable), serving as a penalty coefficient that autonomously adjusts to mitigate potential constraint violations. Thus, the dual problem requires solving the following optimization:

(23) $$\begin{array}{c} \max_{\lambda \geq 0}\min_{\theta }J\left(\theta \right)+\lambda \left(\beta -H\left(\theta \right)\right). \end{array}$$

The primal–dual gradient descent method resolves the optimization challenges presented by alternating updates of policy parameters θ and the Lagrange multipliers λ to achieve optimal solutions. Initially, λ is held constant to solve the minimization problem for θ, expressed as

(24) $$\begin{array}{c} \min_{\theta }J\left(\theta \right)-\lambda H(\theta ). \end{array}$$

Subsequently, with θ fixed, the process maximizes with respect to λ, focusing on

(25) $$\begin{array}{c} \max_{\lambda \geq 0}\lambda \left(\beta -H\left(\theta \right)\right). \end{array}$$

These optimization steps are alternated until convergence, ensuring stability in the values of θ and λ. The updates are carried out as follows:

(26) $$\begin{array}{cc} \theta & \leftarrow \theta -\alpha_{\theta }\nabla_{\theta }L\left(\theta ,\lambda \right),\\ \lambda & \leftarrow \lambda +\alpha_{\lambda }\nabla_{\lambda }L\left(\theta ,\lambda \right), \end{array}$$

where $\alpha_{\theta }$ and $\alpha_{\lambda }$ denote the respective learning rates and $\nabla_{\theta }L\left(\theta ,\lambda \right)$ and $\nabla_{\lambda }L\left(\theta ,\lambda \right)$ denote the gradients of L(θ,λ) with respect to θ and λ. This methodology allows for iterative refinement of the policy during training, integrating maximum entropy to bolster the policy’s exploration capabilities while reinforcing the model’s robustness.

Policy training and evaluation outlines the main details, as shown in the latter part of Algorithm 1. First, all trajectories used for training are extracted from the AIM dataset. From these trajectories, all training samples with a context length of *K* are extracted (lines 18–22). These samples are then trained using the transformer, which predicts the next action based on RTG, CTG, past states, and actions. The NLL loss between the predicted and actual actions is computed, then the entropy of the predicted actions is calculated to construct the Lagrangian function, which is solved using the primal–dual method. This approach continuously updates the model parameters to achieve the goal of training (lines 23–30).

## 5. Experiment

Experiments were conducted in the CARLA high-fidelity autonomous driving simulator [41] to evaluate the performance of the proposed method. This section first provides a detailed description of the parameters and specific experimental settings used during implementation, then analyzes the training process and presents the results obtained from testing the trained model upon deployment.

### 5.1. Experimental Settings

All experiments were conducted in a simulated environment. A road intersection scenario was built using CARLA 0.9.11, and the RL model was constructed based on the PyTorch framework. Additionally, CARLA’s built-in sensors were used to transmit vehicle state information in real time. The initial positions and speeds of all vehicles were randomly set, and traffic operated in a continuous framework rather than a segmented one. The desired vehicle speed and steering angle predicted by the policy neural network were converted into control signals for longitudinal (throttle and brake) and lateral (steering angle) motion using PID controllers. This integration ensured precise maneuverability and stability of the vehicles in the simulated environment. The experiments were conducted on an NVIDIA GeForce RTX 3090 GPU with Ubuntu 18.04 as the operating system. The total time spent on interacting with the environment to collect datasets and training the model was approximately 70 h.

This experiment used a four-way dual-lane unsignalized intersection in CARLA TOWN 05 to train and test the RL model. The road width was 14.2 m. Considering the road characteristics in the CARLA map and the V2I communication range, the control distance was set to 70 m in both the east–west and north–south directions. Vehicle lengths ranged from 3.6 to 5.4 m, widths from 1.8 to 2.2 m, and heights from 1.5 to 2 m, with vehicle arrivals following a Poisson distribution. Consistent with the actual vehicle control cycle, the time step was set to 0.1 s. This simulation environment was meticulously constructed to reflect real-world intersection scenarios in order to ensure the model’s performance and applicability under actual traffic conditions.

This section primarily covers two sets of experiments. The first set evaluated the performance of CoBT-SRL during training, including key metric changes and convergence speed. The second set compared the test results of the trained policy in terms of traffic efficiency, driving safety, and passenger comfort. The main hyperparameters of the experiments are shown in Table 1.

In order to comprehensively evaluate the proposed method, CoBT-SRL was compared with existing centralized coordination methods for unsignalized intersections. The benchmark methods included:

(1) PPO [15]: This centralized coordination scheme for autonomous vehicles utilizes an MA-PPO algorithm, integrating a priori models into PPO to enhance sample efficiency and accelerate the learning process. This method achieves outstanding performance in both traffic efficiency and computation time. The state space includes information on each vehicle’s distance to the center of its corresponding trajectory and its velocity, resulting in a total of sixteen dimensions. The action space consists of the longitudinal acceleration for each vehicle, comprising eight dimensions. The reward function is designed to prioritize traffic safety and improve efficiency while encouraging task completion. A high negative reward is assigned for collisions, a small negative reward is given at each step to minimize waiting time, a positive reward is provided when a vehicle successfully passes through the intersection, and a higher positive reward is granted when all vehicles safely pass through the intersection.
(2) TD3 [17]: A deep reinforcement learning policy based on the TD3 algorithm is employed to optimize cooperative trajectory planning at unsignalized intersections. This approach improves traffic throughput and safety for controlled vehicles (CVs) while reducing computational complexity. The state space includes the position and velocity of each CV, their intended maneuvers (left turn, straight, or right turn), and the queue states, providing a comprehensive view of the traffic situation. The action space comprises acceleration commands that dictate the vehicles’ velocity, heading angle, and steering angle. The reward function balances safety and efficiency. Large negative rewards are assigned for collisions, while positive rewards are given for vehicles that pass through the intersection without incidents. Additionally, intermediate rewards are granted to encourage the completion of maneuvers, with overall system performance evaluated by summing individual rewards.
(3) VICS [10]: This centralized coordination method for unsignalized intersections utilizes an MPC framework to optimize vehicle trajectories, aiming to prevent collisions and enhance traffic efficiency. The state space comprises the position, velocity, and acceleration of each vehicle approaching the intersection, represented in a Cartesian coordinate system. The control inputs, which are the longitudinal accelerations of the vehicles, are optimized to ensure efficient and safe passage through the intersection. The objective function is designed to minimize the risk of cross-collisions by controlling vehicle trajectories, considering constraints such as maximum velocity, safe following distance, and lateral acceleration limits for turning maneuvers. This optimization problem is addressed iteratively, with control inputs recalculated at each time step to adapt to real-time traffic conditions and ensure collision-free operation.

The training time for PPO was approximately 120 h, while the training time for TD3 was around 80 h. In contrast, VICS employed the MPC method for control, which did not require any training. All these methods were trained under the same hardware environment to ensure fairness and comparability of the experimental results.

### 5.2. Training Performance

**Figure 4:**
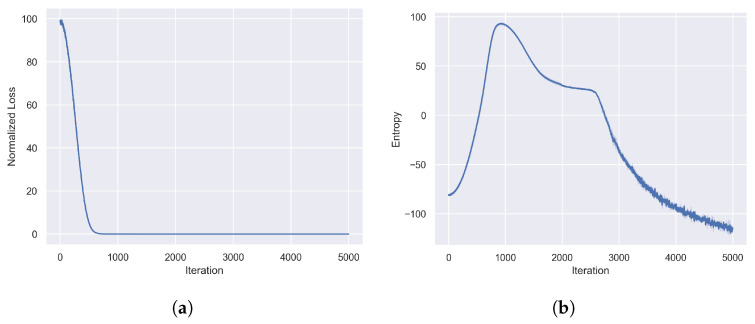
Performance evaluation during training: (**a**) normalized loss function and (**b**) exploration entropy.

In this experiment, CoBT-SRL was trained on the expert dataset according to the parameters set in Table 1; the training results are shown in Figure 4. Figure 4a depicts the variation of the loss function during training. As shown, the loss value converged before 1000 iterations and remained stable thereafter, indicating a rapid convergence speed of CoBT-SRL during the training process. Figure 4b illustrates the change in exploration entropy throughout the training process. The exploration entropy increased during the initial training phase, peaked around 1000 iterations, and then gradually decreased. The rate of decrease was slow between 1000 and 2500 iterations, accelerated after 2500 iterations, and eventually converged to a low level. This indicates that CoBT-SRL’s exploratory nature initially increased and then decreased before ultimately converging to a lower level. This helps the model better explore the environment during training, enhancing its generalization performance.

During the experiment, the loss function showed a rapid decline in the initial phase and stabilized after 1000 iterations, indicating that the model quickly adjusted its parameters to fit the training data. This rapid convergence not only shortens the training time but also provides a stable foundation for subsequent model tuning, demonstrating the algorithm’s efficiency during learning.

Simultaneously, the exploration entropy curve reveals the model’s ability to explore the policy space. The initial rise in entropy indicates the model’s high tolerance for environmental uncertainty, encouraging diverse policy exploration. After peaking, the entropy gradually decreased as iterations progressed, with a more rapid decline after 2500 iterations. This trend suggests that after sufficiently exploring various policy, the model gradually focuses on optimizing the existing policy, thereby improving overall performance. This shift from exploration to exploitation ensures that the model not only thoroughly explores the policy space but also effectively selects the optimal policy to enhance generalization and adaptability.

### 5.3. Testing Performance

The trained CoBT-SRL model was tested and compared with the aforementioned benchmark methods. To comprehensively evaluate the performance of CoBT-SRL and other methods, tests were conducted in unsignalized intersection scenarios under both sparse and dense traffic conditions. The primary evaluation metrics included average travel time, collision rate, TTC violations, and average acceleration. Average travel time was used to assess traffic efficiency, collision rate and TTC violations were used to evaluate driving safety, and average acceleration was used to measure passenger comfort. The test results are shown in Table 2. From the table, it is clear that CoBT-SRL achieved the best performance under both sparse and dense traffic conditions, with collision rates and TTC violations both at 0, average travel times of 16.34 s and 21.91 s, and average accelerations of 0.39 m/s^2^ and 0.30 m/s^2^, respectively. Compared to other benchmark methods, CoBT-SRL demonstrated superior performance in traffic efficiency, driving safety, and passenger comfort, indicating significant advantages in coordinating autonomous vehicles at unsignalized intersections. Although the VICS method based on MPC also achieved a zero collision rate, it performed less well in terms of traffic efficiency and passenger comfort, highlighting the distinct advantages of CoBT-SRL.

**Figure 5:**
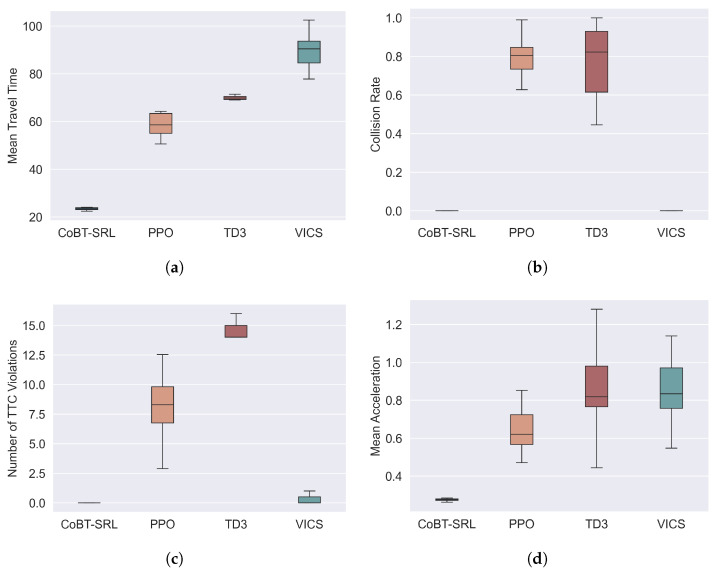
Performance comparison after deployment in terms of efficiency, safety, and comfort: (**a**) mean travel time; (**b**) collision rate; (**c**) number of TTC violations; (**d**) mean acceleration.

Additionally, to evaluate the distribution and stability of the test data from multiple runs, box plots were used to represent the test results. Using the dense traffic scenario as an example, the test results are shown in Figure 5. Figure 5a shows the distribution of the average travel time. From the figure, it can be seen that CoBT-SRL’s average travel time is relatively stable and short, indicating an advantage in traffic efficiency. The average travel times of the other three methods are higher than that of CoBT-SRL, with VICS having the longest average travel time due to its optimization-based approach, which requires longer computation times. Although TD3 also has a low standard deviation in average travel time, its mean value is much higher than CoBT-SRL, demonstrating CoBT-SRL’s superior performance in traffic efficiency.

Figure 5b shows the distribution of collision rates. From the box plot, it can be seen that both CoBT-SRL and VICS have collision rates of 0, while PPO and TD3 have higher collision rates. VICS achieves a collision rate of 0 because its MPC-based method can optimize vehicle trajectories through optimal control to avoid collisions. Similarly, CoBT-SRL achieves a collision rate of 0 by considering safety constraints during training, effectively preventing collisions. In contrast, PPO and TD3, which do not prioritize safety constraints, have higher collision rates, highlighting CoBT-SRL’s significant advantage in driving safety.

Figure 5c shows the distribution of TTC violations. It can be seen that CoBT-SRL has zero TTC violations, while VICS also has a low level of TTC violations. Similar to VICS, CoBT-SRL can optimize vehicle trajectories through optimal control to avoid TTC violations, demonstrating excellent performance in ensuring driving safety. In contrast, PPO and TD3 have a higher number of TTC violations, mainly because these RL methods do not prioritize safety constraints, further underscoring CoBT-SRL’s superior results in driving safety.

Figure 5d shows the distribution of average acceleration. CoBT-SRL’s average acceleration is relatively stable and low, while the average acceleration of the other three algorithms is higher and shows a more unstable overall distribution. Average acceleration reflects passenger comfort, with smoother driving improving passenger comfort. Therefore, the lower acceleration of CoBT-SRL indicates an advantage in passenger comfort.

In summary, CoBT-SRL achieves the best performance in traffic efficiency, driving safety, and passenger comfort, demonstrating significant advantages in coordinating autonomous vehicles at unsignalized intersections. Additionally, the proposed algorithm demonstrates a certain level of generalizability, making it applicable to other types of intersection scenarios, such as three-way intersections (T-junctions or Y-junctions), roundabouts, and other irregular traffic environments. Before applying the algorithm to new settings, it is essential to fine-tune the model’s hyperparameters, including adjusting the coefficients of the reward and cost functions, vehicle turning formulas, control area size, and other environment-specific parameters. For scenarios with significantly increased complexity, additional state variables such as heading angle, driving direction, etc., can be introduced to provide a more comprehensive description of the traffic situation. Furthermore, more action options, such as passage permissions, route choices, etc., can be included to optimize traffic flow control. Increasing the number of neural network layers and neurons per layer can enhance the model’s flexibility to handle more complex problems, although it is important to consider the risk of overfitting that may arise from a more complex model. Therefore, with careful design and appropriate optimization, the proposed method is expected to deliver excellent performance even in more complex situations.

## 6. Conclusions

This study proposes the CoBT-SRL framework for centralized coordination among vehicles at unsignalized intersections, aiming to address the limitations of traditional RL policy networks in reasoning capability and ensuring policy safety. First, an expert dataset is constructed by collecting data on past states, actions, RTG, and CTG, and data filtering is performed. Leveraging the powerful ability of transformers to capture long-distance dependencies when solving sequence modeling problems, autoregressive modeling is applied to the collected dataset to predict future actions based on past states, actions, and other trajectory information. Second, the proposed framework introduces a maximum-entropy RL approach, which increases policy exploration by maximizing the entropy of the policy within constrained bounds, thereby enhancing the model’s generalization performance. Experimental results demonstrate that CoBT-SRL offers significant advantages in centralized coordination of autonomous vehicles at unsignalized intersections, exhibiting excellent traffic efficiency, driving safety, and passenger comfort.

Although CoBT-SRL achieves significant advantages in centralized coordination of autonomous vehicles at unsignalized intersections, several challenges remain when applying this technology to more complex traffic scenarios. The current research primarily focuses on the management of single intersections. Extending this approach to broader traffic systems, such as coordinated control between main roads or collaborative management of multiple intersections within the same network, holds more profound practical significance and application value. Moreover, in light of the rapid development and widespread application of large models, future research should explore how to further leverage these advanced technologies. Through this approach we aim to significantly enhance the model’s generalization capability and reasoning performance, enabling more effective handling of complex and dynamic traffic environments.

## Figures and Tables

**Table 1:** Hyperparameters for experiments.

Parameters	Value
CARLA Simulator	
Road width *W*	14.2 m
Timestep *t*	0.1 s
Length of control distance	70 m
**CoBT-SRL**	
Training context length *K*	10
Number of layers $n_{l}$	4
Attention heads $n_{l}$	4
Target entropy β	−80
Embedding dimension	512
Dropout	0.1
Learning rate	$1\times 10^{-4}$
Weight decay	$1\times 10^{-3}$
Batch size	256
Reward and cost parameters $\delta_{1},\delta_{2},\delta_{3},\delta_{4}$	5, 0.6, 0.6, 100
Reward weights $\omega_{1},\omega_{2},\omega_{3}$	0.05, 0.1, 0.1
**PPO**	
Learning rate	$3\times 10^{-4}$
Clip ratio	0.2
Minibatch size	64
**TD3**	
Learning rate	$3\times 10^{-4}$
Soft update rate	$5\times 10^{-3}$
Exploration noise	0.1
Policy noise	0.2
Clip ratio	0.5
Batch size	256
Policy delay	2
**VICS**	
Predictive horizon *T*	5
Weights $\omega_{v},\omega_{a},H$	1, 5, 1000
Risk parameter α	0.005

**Table 2:** Testing performance comparison.

Traffic Density	Metrics	CoBT-SRL	PPO [15]	TD3 [17]	VICS [10]
Sparse	Coll Rate (%)	0.00	64.26	66.73	0.00
	TTC Viol (-)	0.00	3.73	9.63	0.69
	Trav Time (s)	18.91	55.69	59.66	82.26
	Accel (m/s^2^)	0.34	0.72	0.93	0.83
Dense	Coll Rate (%)	0.00	77.99	69.86	0.00
	TTC Viol (-)	0.00	7.95	12.78	0.79
	Trav Time (s)	23.25	61.70	65.30	90.37
	Accel (m/s^2^)	0.31	0.65	0.88	0.86

## Data Availability

The data presented in this study are available upon request from the corresponding author.
